# Mammary Abscess

**Published:** 1859-01

**Authors:** A. B. Crosby


### SELECTIONS.

*Mammary Abscess.* By A. B. CROSBY, M. D.

Chassaignac and other authors have divided and subdivided abscess of the mamma, almost infinitely. Such classifications, while they do great credit to the ingenuity and accuracy of observers, are practically of less value than more simple arrangements. We shall consider this affection as occurring under two forms only, viz; extra and intra mammary abscess.

Under the head of extra mammary abscess, we shall consider all those deposits of pus which may occur external to the gland, or to any part of it.

Intra mammary abscess will cover inflammation and suppuration of the substance of the gland.

*Causes.*—A great variety of causes may give rise to this form of abscess.

Sometimes stimulating food is resorted to immediately after parturition, and the quantity taken large; this may occasion inflammation of the breast. Over anxiety on the subject, and the injudicious use of cold water have been observed as causes. Mechanical injuries of the breast not unfrequently give rise to inflammation. Suppuration is more likely to occur in women who do not suckle their children, or who wean them too early. At times irritation and congestion occur, when the child is first put to the breast, and unless relieved, abscess is almost certain to occur. The milk may be retained in the organ, and induration reaching the axilla may be followed by suppuration. Too much exercise of the arms after parturition, or irritation of the womb, may either of them prove a sufficient cause.

This form of abscess is not confined to the period of pregnancy nor lactation, but may occur at any time. Thus injury of the breast or dyscrasia, may give rise to it at any period. Persons tainted with scrofula, gout or syphilis are far more liable than those who have no hereditary predisposition to such affections.

*Symptoms.* In extra mammary abscess the swelling is usually uniform, whether it be large or small. If it is superficial, the skin is smooth, red and shiny, and the pain more or less severe. The secretion of milk may or may not cease. When the abscess is intra mammary, the swelling is much harder and not so uniform. The surface of the swelling is tabulated and the pain more intense. In the early stage the secretion of milk is suspended, but later in the course of the affection it is renewed again. When extra mammary inflammation occurs, its most common termination is more frequently followed by suppuration.

The ease with which mammary abscess will be detected must depend on the depth at which it occurs, and the positive or negative character of the symptoms.

Constitutional signs are at times slight, at other times they may be of the most grave character. The pulse is accelerated, and inflammatory in its character. The tongue is heavily furred, and there is violent headache.

The febrile symptoms are well marked, and urine is scanty and high colored.

Sometimes the inflammation occurs deep, seated beneath the breast and the term submammary, has been applied to it. In such cases the pain is deep seated and the swelling colorless. Motion of the arm is attended with an increase of pain, and suppuration occurs slowly.—The pus finds its way to the surface after a considerable time has elapsed. It may point at one place or at several. Sometimes the pus forms and remains quiescent for a long time The swelling of such a collection has been mi taken for a malignant tumor and its extirpation attempted. Suppuration of the breast may occur in from four to ten days. The pus may then approach the surface by ulceration.—These processes are indicated by shivering, more or less heat ensues, which is liable to be followed by a profuse diaphoresis. The difficulty of detecting fluctuation will be directly proportioned to the depth of the abscess. Pointing may, and frequently does, occur about the nipple. The tumor is smooth and prominent and may point in several places.

If the inflammation has been superficial, opening occurs spontaneously and the discharge usually consists of healthy pus. If, however the seat of the abscess is deep, and considerable time elapses before its contents reach the surface, extensive sloughs of fascia and areolar tisues are liable to form.

Even after one of these abscesses has terminated favorably, induration of the breast may remain for a considerable period of time.

In persons of a scrofulous habit, deep seated abscess of the breast not infrequently proves fatal. Rigors occur daily, and these are followed like intermittent, by a season of fever, and then of profuse sweating. Induration of the breast takes place or sinuses may form in different directions through the organ. There is an entire loss of appetite and the patient suffers from a persistent nausea.

The progress of the abscess is slow, and if not interfered with, its contents at length approach the surface. After this, the symptoms abate and there is an apparent improvement in the patient's condition. This, however, is only temporary, and the unfavorable signs return more intractable than ever. It is probable that a secondary deposit of pus has occurred. Not unfrequently some point may be discovered on the breast, which is œdematous, and a careful examination may indicate obscure fluctuation. If free exit is not given to this pus, very great exhaustion results. Sometimes openings need to be made at different points repeatedly, and if neglected, sinuses are formed, which contain an extremely fœtid pus. The other breast may now take on inflammation, accompanied by signs of exhaustion. Nausea and shivering are quite constant. The patient vomits bilious matter and loaths food entirely. The skin is either dry and feverish or cool and clammy. The mesenteric glands are liable to become diseased and the uterus, too, becomes affected, and a discharge is established from the vagina. The pulse frequent and feeble, exhaustion rests upon the sufferer like an incubus, and death finally results from asthenia.

Such are some of the signs of abscess of the breast, which vary with the habit, the person and the cause.

*Treatment.* The first indication in the treatment is, if possible, to abort the inflammation.

Venesection may sometimes be resorted to with advantage, especially when the constitutional symptoms are violent; generally, however, this will be unnecessary. Leeches may be applied to the inflamed breast with benefit, and may be repeated if occasion requires.

Emollient applications may be made and if there is much heat, an evaporating lotion. Warm vinegar and opium relieve the pain and answer a good purpose.

In the early stages, the hardness of the breast may sometimes be wholly discussed by gentle friction with camphorated oil or some anodyne liniment.

Saline purgatives should be resorted to early and freely. But the remedy which is most efficacious, is antimony. This drug may be exhibited in doses of one sixteenth of a grain and continued until the desired effect is produced.

It is not advisable to excite vomiting, but simply to push the remedy to the verge of nausea. By the use of this agent we may often succeed in diminishing the force of the circulation and aborting the inflammation at once. It seems to exert almost a specific action on the mammæ, and is the remedial agent on which most dependence can be placed.

The diet during this early stage should be bland, unstimulating and mostly fluid. The breast should be supported by a bandage to prevent it from depending too much, and the milk may be drawn gently, and at suitable intervals.

The second indication in the treatment is to promote suppuration. Poultices accomplish this best, and they should be generous and frequently renewed. The different preparations of opium may be resorted to for the relief of pain, and saline cathartics as the case may require.

When suppuration has occurred the question arises whether the abscess be allowed to burst, or whether it shall be opened. Different authors have maintain different positions on this question. Sir Astley Cooper contended that they should be opened and the weight of authority is with him.

If the abscess is superficial it may do no harm if it is allowed to burst. Generally, and especially if the collection is deep seated, the knife should be resorted to as soon as the pus can be detected. It may be necessary to introduce a tent, and renew it from time to time until the cavity is obliterated.

If the abscess has been small, or if milk is not discharged with the pus, the child may be allowed to nurse moderately from the affected breast.

Sometimes the suckling excites inflammation in the opposite breast. When this is the case the child should be removed from the breast altogether. Even if there is nothing about the mother to contra-indicate nursing, and yet the child seems unwell, it is better to feed it.—Generally if there is pus in the milk, the child will not nurse, so that nature will almost always indicate whether the nursing should continue or not.

More or less debility accompanies mammary abscess and may prove the source of greatest danger. This condition is best combated by tonics, and in the later stages by a generous diet.

At times the abscess will not heal, and a chronic discharge occurs. The application of adhesive strips so as to make gentle pressure, serves to do away this unfavorable condition.

If sinuses occur they are to be laid open freely with the knife, when they usually heal.

Dr. S. C. Foster of New York has published an article in which he takes the ground that mammary abscess may and should be prevented. The exceptions to this rule are cases in which there is a taint of syphilis or scrofula. Mechanical injury, obstruction of the nipple, and an endemic tendency to purulent deposits, may also give rise to the affection beyond the power of prevention.

His views are in brief something like the following:—

Prevention of mammary abscess should be commenced at puberty. The dress should be loose, and the young girl should take an abundance of exercise. Lacing has a tendency to depress the nipple, and so lay up much suffering for the future mother. If This has occurred the nipple should be gently drawn out during pregnancy, and rendered hard by the application of borax and brandy. Some caution is necessary, or the breast may be so far stimulated as to induce premature labor.

In the early stage of mammary abscess accumulation of milk is to be guarded against by gentle but firm friction extending from the periphery towards the nipple. If this is done thoroughly the induration may frequently yield in a few minutes.

If this is not successful, compressed sponge may be employed either during the stage of induration or after pus has formed and escaped A moist sponge is to be subjected to a power press for twenty-four hours or until it is dry. Such a sponge is to be applied to the part of the organ affected, retained in its position by a roller and then thoroughly moistened with tepid water.

It is claimed for the sponge that it exerts a uniform, gentle pressure, is emollient like a poultice, absorbs milk and pus, and promotes their flow by capillary attraction.

The simplicity of this method of treatment, as well as the favorable character of its recorded results, entitle it to respect and a fair trial.

Chassaignac advises the injection of the cavity of the abscess with tepid water, and the application of a cup until it is entirely emptied. Adhesive strips are then applied so as to make a firm but gentle pressure. If this treatment occasions any irritation the dressing is to be removed, and reapplied as circumstances may indicate.

It is claimed for both of these methods that recovery will take place in from one to eight days.—*N. H. Journal of Medicine.*

